# Electron Transfer in a Bio-Photoelectrode Based on Photosystem I Multilayer Immobilized on the Conducting Glass

**DOI:** 10.3390/ijms23094774

**Published:** 2022-04-26

**Authors:** Sebastian Szewczyk, Alice Goyal, Mateusz Abram, Gotard Burdziński, Joanna Kargul, Krzysztof Gibasiewicz

**Affiliations:** 1 Faculty of Physics, Adam Mickiewicz University in Poznań, ul. Uniwersytetu Poznańskiego 2, 61-614 Poznań, Poland; sszew@amu.edu.pl (S.S.); alice.goyal@thapar.edu (A.G.); gotardb@amu.edu.pl (G.B.); 2 Solar Fuels Laboratory, Centre of New Technologies, University of Warsaw, Banacha 2C, 02-097 Warsaw, Poland; m.abram@cent.uw.edu.pl; 3 Faculty of Biology, University of Warsaw, Miecznikowa 1, 02-096 Warsaw, Poland

**Keywords:** photosystem I, time-resolved absorption spectroscopy, chronoamperometry, biohybrid photoelectrodes, electron transfer, photoelectrochemical cell

## Abstract

A film of ~40 layers of partially oriented photosystem I (PSI) complexes isolated from the red alga *Cyanidioschyzon merolae* formed on the conducting glass through electrodeposition was investigated by time-resolved absorption spectroscopy and chronoamperometry. The experiments were performed at a range of electric potentials applied to the film and at different compositions of electrolyte solution being in contact with the film. The amount of immobilized proteins supporting light-induced charge separation (active PSI) ranged from ~10%, in the absence of any reducing agents (redox compounds or low potential), to ~20% when ascorbate and 2,6-dichlorophenolindophenol were added, and to ~35% when the high negative potential was additionally applied. The origin of the large fraction of permanently inactive PSI (65–90%) was unclear. Both reducing agents increased the subpopulation of active PSI complexes, with the neutral P700 primary electron donor, by reducing significant fractions of the photo-oxidized P700 species. The efficiencies of light-induced charge separation in the PSI film (10–35%) did not translate into an equally effective generation of photocurrent, whose internal quantum efficiency reached the maximal value of 0.47% at the lowest potentials. This mismatch indicates that the vast majority of the charge-separated states in multilayered PSI complexes underwent charge recombination.

## 1. Introduction

**Figure 1:**
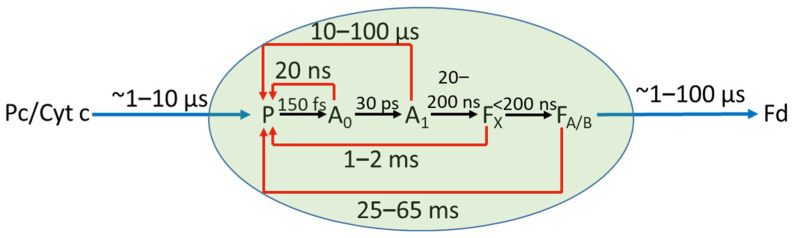
Scheme of ET reactions inside PSI complex and between PSI and natural external electron donor (plastocyanin, Pc, or cytochrome *c*, Cyt C) and acceptor (ferredoxin, Fd). Forward ET reactions are represented by black arrows and are completed (the state P^+^F_A/B_^−^ is formed) in a submicrosecond time range [8]. Backward ET reactions are represented by red arrows, and ET between PSI and external carriers—by blue arrows. For simplicity, the two branches of initial ET cofactors were lumped together into one. Moreover, two final electron acceptors, F_A_ and F_B_, are lumped together into one, F_A/B_.

When searching for novel clean sources of energy, inspiration is often gained from natural light-harvesting macromolecular systems. Photosystem I (PSI) is one of the photosynthetic pigment–protein complexes, present in higher plants, algae, and cyanobacteria, which is often used as a light-sensitizing material in the proof-of-concept biohybrid photoelectrodes [1,2,3,4,5,6,7]. This is rational, since native PSI is a robust and abundant protein that generates substantial electric potential of 1 V and electric current with almost 100% quantum yield of the photon-to-photoelectron conversion [8,9,10,11]. Moreover, PSI complexes isolated from the membranes of extremophilic phototrophs, e.g., red alga *Cyanidioschyzon merolae* (*C. merolae*), are resistant to harsh environmental conditions, such as high temperature (up to 80 °C), extreme pH (pH 4–12), and extremely high solar irradiance (up to 25,000 μE/m^2^/s; [12]). An important feature of PSI complexes, along with other photosynthetic reaction centers, which ensures their exceptionally high efficiency, is that the light-induced charge separation, i.e., electron transfer (ET) from the excited primary electron donor to a chain of redox active cofactors, occurs inside these proteins in an almost irreversible way [8,9]. This is because the forward ET reactions occur much faster than the back ET or charge recombination (Figure 1). In the natural, fully functional photosynthetic system, after the excited electron from the PSI primary donor (a chlorophyll *a* dimer, P) reaches the final PSI acceptors (iron–sulfur clusters, F_A/B_), the PSI complex is recovered by the electron donation to P^+^ from a freely defusing water-soluble copper–protein complex (plastocyanin) or heme-containing cytochrome (*c*_6_ or its homologue, *c*_553_, in the case of *C. merolae*). Concomitantly, electron uptake from F_A/B_^−^ by the ferredoxin metalloprotein occurs on the opposite side of the membrane (Figure 1). These two ET reactions occur in the natural system within 1–12 μs [13,14,15,16] and in the submicro- to microsecond time regime [17,18,19,20,21], respectively. These ET reactions are much faster than P^+^F_A/B_^−^ → PF_A/B_ recombination, whose lifetime is 25–65 ms [22]. The charge recombination reactions were investigated in PSI complexes isolated from photosynthetic membranes, and thus were devoid of natural electron donor (plastocyanin/cytochrome *c*) and acceptor (ferredoxin) [22,23,24]. In addition to the P^+^F_A/B_^−^ recombination, faster phases of recombination were also detected in these studies, in particular, for PSI complexes intentionally disturbed, and assigned to charge recombination between P^+^ and intermediate PSI electron acceptors: iron–sulfur cluster, F_X_^−^, phylloquinones, A_1_^−^, and chlorophylls, A_0_^−^. The lifetimes of the respective reactions were 1–2 ms (P^+^F_X_^−^ → PF_X_), 10–100 μs (P^+^A_1_^−^ → PA_1_), and 20 ns (P^+^A_0_^−^ → PA_0_) (see Figure 1).

When incorporating isolated PSI particles into an artificial light-conversion system, a few issues are of particular importance in order to utilize their full functionality [25]. The first of them is to which extent the natural ET properties are preserved inside the PSI complex after its incorporation into the artificial system. Another one is the efficiency of coupling of the donor and acceptor sides of PSI with artificial electron carriers, which replace natural plastocyanin/cytochrome *c* and ferredoxin. Regarding the first issue, it was shown that embedding PSI particles in the trehalose glass matrix leads to the partial inhibition of forward ET. The fractions of PSI complexes in which ET did not proceed beyond A_0_, A_1_, and F_X_ were 53%, 16%, and 22%, respectively [22]. In consequence, only ~10% of electrons reached the terminal F_A/B_ acceptors. This result demonstrates that the small efficiencies of photocurrent generation in the semi-artificial biohybrid devices may be due to the limited forward ET inside immobilized complexes, followed by internal charge recombination. Indeed, very limited internal quantum efficiencies (IQE, defined as a ratio of the number of photocurrent electrons efficiently generated in the system to the number of absorbed photons) of PSI-based photoelectrodes were reported ([26]—5%, [27]—0.37%). More often, only absolute photocurrents were reported in the literature, and the light conversion efficiencies were not [28,29,30,31,32]. On the other hand, the reported efficiencies of PSI-based photoelectrodes are sometimes substantial [33] but the full functionality of the photosynthetic reaction centers was not convincingly confirmed in these studies [34,35].

An approach proposed in the present study is to correlate the results of photocurrent efficiency obtained for the PSI-based photoelectrode with the results of the transient absorption measurements of ET kinetics inside PSI deposited on the electrode. In principle, this approach may help to assess to what extent the limitation of the photocurrent generation is due to the disturbance of ET in PSI (or other reaction centers used instead of PSI), or, oppositely, it may demonstrate that the photocurrent generation efficiency is high in a specific device despite disturbance of ET in the protein, thus indicating a different source of photocurrent generation than the native PSI complex. An obvious condition for this approach to be applicable is that the PSI-based photoelectrode must be at least partially transparent.

Here, we have studied a simple photoelectrode composed of a multilayer of PSI particles isolated from the *C. merolae* microalga and electrodeposited directly onto the fluorine tin oxide (FTO) conducting glass. A similar photoelectrode was shown to be characterized by a small IQE value of 0.26% despite almost full charge separation (formation of the P^+^F_A/B_^−^ state with 90% efficiency) in these PSI particles dissolved in an aqueous solution [24]. Previously, it was demonstrated that similar PSI-FTO systems, based on PSI from cyanobacterium *Synechocystis* sp. PCC 6803, were fully functional in terms of excitation energy transfer and the primary charge separation [36]. Consequently, a question emerges whether immobilization of PSI particles on FTO significantly disturbs secondary ET reactions inside the protein, which may result in the low IQE value for the PSI-FTO electrode.

## 2. Materials and Methods

### 2.1. Preparation of PSI Particles

PSI particles were isolated from a thermo-acidophilic red microalga *C. merolae* grown under a high light intensity (CmH; 350 μE m^−2^ s^−1^), as described elsewhere [12,37]. PSI complexes grown under these conditions were composed of the PSI core and the peripheral antenna, light-harvesting complex I (LHCI), containing on average four Lhcr antenna subunits. In consequence, the PSI particles contained ~159 antenna Chls *a* per P [12]. The isolation was performed using n-dodecyl-β-D-maltoside (β-DM) and the proteins were purified by ion exchange chromatography. Isolated complexes were kept frozen in the presence of 20–25% (*w*/*v*) glycerol at –55 °C prior to spectroscopic and electrochemical experiments.

### 2.2. Electrodeposition of PSI on FTO Conducting Glass and Steady-State Absorption Measurements

**Figure 2:**
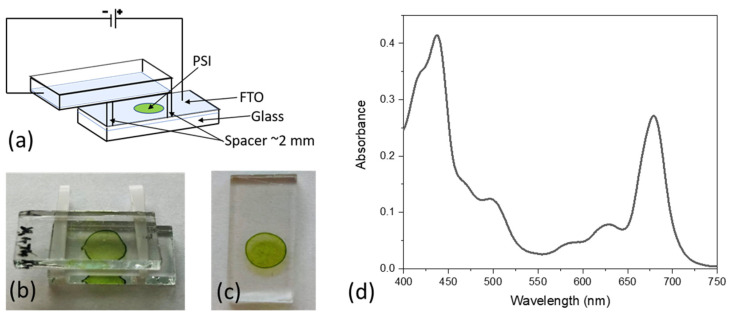
PSI multilayer immobilized on FTO. (**a**) Scheme of the setup for electrodeposition of PSI particles on the FTO conducting glass; (**b**) picture of the setup for the electrodeposition; (**c**) picture of the bio-photoelectrode ready to use with estimated number of ~40 PSI layers; (**d**) absorption spectrum of the bio-photoelectrode shown in panel c.

A detailed procedure of the electrodeposition was described previously [27,36]. Briefly, before immobilization, the PSI solution was dialyzed in distilled water with the addition of 5 mM Bis-Tris buffer (pH 7.0) to reduce the concentration of salts and detergent. Next, a droplet of a volume of 30 µL of dialyzed PSI solution of absorbance A_679nm,1cm_ ≈ 8.5 was placed on one FTO glass electrode and covered with another one. The two FTO electrodes were facing with their conductive sides towards the solution and were separated by a ~2-mm-thick spacer (Figure 2). Next, a potential difference of +2 V between the lower and upper electrodes was applied for 5 min, which caused partial orientation of PSI particles and their migration toward the bottom electrode. After that, the samples were dried at 4 °C overnight and stored at 4 °C prior to measurements. As a result, a multilayer of the area of ~0.25 cm^2^ of partially oriented complexes was formed, with the PSI donor side (P side) being preferentially proximal to the bottom electrode surface and acceptor side (F_A/B_ side)—distal from this surface. The absorption spectrum of the multilayer was measured using a Hitachi U-2800A spectrophotometer and was corrected for the absorption of FTO glass and light scattering by the PSI film (Figure 2d). The absorbance of the dry PSI multilayer at Q_y_ band maximum (~679 nm) was A_679_ = 0.27. The estimated number of PSI monolayers, *n_PSI_*, deposited onto FTO was calculated to be ~40 using the following equation [27]:

(1) $$n_{PSI}=\frac{A_{679}N_{A}d^{2}}{\varepsilon_{679}m},$$

where *N_A_* is the Avogadro number, *d* = 15 nm—diameter of PSI complex, $\varepsilon_{679}$≈ 57 000 M^−1^cm^−1^—molar extinction coefficient of Chl *a* in PSI [38], and *m* = 159 is the number of Chls *a* per *C. merolae* PSI complex [12]. The estimated thickness of the PSI film was 0.6 μm.

### 2.3. Photocurrent Measurements

**Figure 3:**
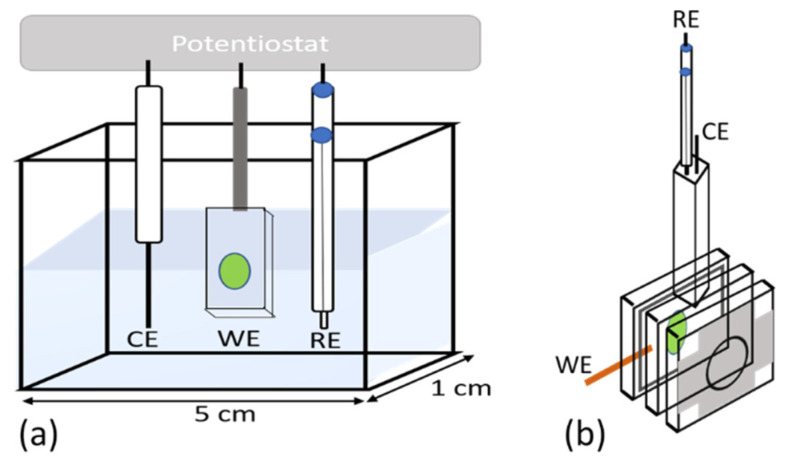
Schemes of the three-electrode photoelectrochemical cells used for: (**a**) photocurrent measurements; (**b**) time-resolved absorption measurements. WE—working electrode, RE—reference electrode, CE—counter electrode.

Photocurrent measurements were performed in a custom-made three-electrode photoelectrochemical cell (PEC) enclosed within a black box with a hole for illumination (Figure 3a; for details, see [27]). The working electrode (WE) was a PSI-FTO plate (Figure 2c), the counter electrode (CE) was a platinum wire, and the reference electrode (RE) was Ag/AgCl electrode (+220 mV vs. standard hydrogen electrode, SHE). The electrodes were connected to the Autolab PGSTAT204 potentiostat. The electrolyte was an aqueous solution of 30 mM Bis-Tris buffer (pH 7.0), 10 mM sodium ascorbate (sacrificial electron donor to P^+^ [3,39]), and 200 µM dichlorophenolindophenol (DCPIP; mediator reduced by the excess of ascorbate). The PSI-FTO working electrode was illuminated with the 685 nm LED (~24-nm spectral bandwidth (FWHM) and 5.8 mW/cm^2^ light power density). The chronoamperometric data, i.e., WE-CE current vs. time, were collected at different potentials applied to the WE, ranging from −280 to +620 mV vs. SHE. Each time after the potential was changed, the dark current was stabilized for ~5 min. At each potential, the dark current “baseline” was subtracted from the raw chronoamperometric data to obtain the effective photocurrent data. The sequence of the applied potentials was kept the same as in the transient absorption measurements (see below). Open circuit potential (OCP), i.e., the potential at which no electrical current flows between WE and CE in the darkness, was measured to be +115 mV vs. SHE. Hereafter, all the potentials are presented in mV vs. SHE. The values of IQE of the PSI-FTO electrode were estimated, as described previously in [27].

### 2.4. Time-Resolved Absorption Measurements

For time-resolved absorption measurements, a specially designed PEC was used (Figure 3b; for details, see [27]) in which PSI-FTO WE was one of the walls, with PSI multilayer being deposited on the internal side of this wall, in contact with the electrolyte solution. Despite different construction, better suited for optical measurements, the overall operation of this PEC was the same as the PEC for chronoamperometric experiments (Figure 3a). The ET kinetics in PSI immobilized on the conducting glass was measured using the transient absorption setup described in [40]. Briefly, the excitation source, Q-switched Nd:YAG laser (532 nm, 8 ns FWHM, 1 mJ per pulse), operated at a repetition rate of 10 Hz. Shutter was used to decrease excitation pulse repetition reaching the sample down to 0.5 Hz or 0.05 Hz. Probe pulse had twice higher frequencies. The absorbance difference signal was calculated from the formula:

ΔA(t) = log (I_exc_(t)/I(t)), (2)

where I_exc_(t) and I(t) are the intensities of the probe light leaving the excited and nonexcited sample, respectively. The configuration of excitation and probe beams was almost antiparallel (close to 180 degrees). The probing light source was a 150-watt xenon arc lamp used in a continuous mode, equipped with a ~700-nm interference filter (10 nm FWHM, FB700-10 Thorlabs) and a shutter, which minimized the actinic effect of the probe light. The diameters of the pump and probe beams were 5 mm and 3 mm, respectively. The probe light was dispersed using a monochromator, which was equipped with an R928 Hamamatsu photomultiplier further connected to a digital oscilloscope. The time window of the experiments was 54 ms.

The kinetic traces were collected at different compositions of the electrolyte (either pure 30 mM Bis-Tris buffer (pH 7.0) or supplemented with 10 mM sodium ascorbate and 0–200 μM DCPIP) at different potentials applied to PSI-FTO WE, ranging from −280 to +620 mV, and at two frequencies of repetition (0.5 Hz and 0.05 Hz). Each time after the potential was changed, a ~5-min interval was given for the system to adapt to the new potential. Each analyzed kinetic curve was an average of 50 individual traces. The averaged curves were fitted with a sum of two exponentials and an offset using the OriginPro software according to the formula:

(3) $$\Delta A\left(t\right)=\sum_{i=1}^{n}A_{i}e^{-\frac{t}{\tau_{i}}}+const,$$

where *τ_i_* is the lifetime of the *i*-th component, *A_i_* is the amplitude associated with this lifetime, and *const* is the amplitude of the component nondecaying on the 54-ms time scale of the experiment.

## 3. Results and Discussion

### 3.1. Photocurrent Measurements

**Figure 4:**
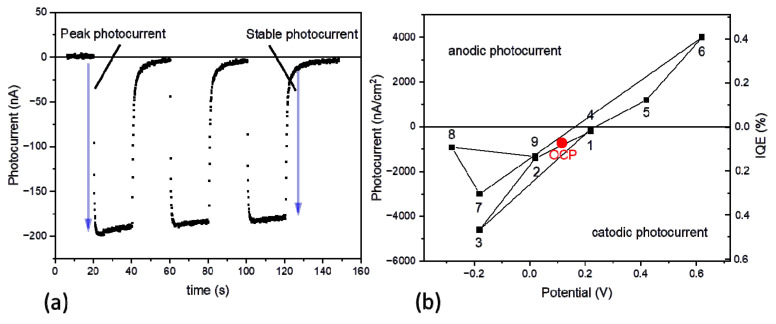
Photocurrent generated in the PSI-FTO electrode with ~40 layers of PSI complexes. (**a**) Chronoamperometric data recorded for the PSI-FTO electrode of the photoactive surface of ~0.25 cm^2^ at OCP (see corresponding red circle in panel b); three cycles of light illumination are presented with peak and stable photocurrents indicated; (**b**) stable photocurrent amplitudes (left axis) and corresponding IQE (right axis) vs. potential applied to WE (dark current baseline has been subtracted from the original chronoamperometric data); the numbers at the experimental points show the sequence of the applied potentials; additionally, photocurrent measured at OCP is shown (red circle). Note that, in panel b, photocurrent was recalculated per centimeter squared.

Figure 4a shows the results of chronoamperometric measurement performed using the PSI-FTO electrode at OCP = +115 mV. The value of the stable photocurrent, ~(–180 nA), corresponds to IQE = 0.073%, which is ~3.5 times lower than the value 0.26% reported for a similar *C. merolae* PSI-FTO electrode [24] but with a much thinner PSI multilayer (A_679_ = 0.03 vs. 0.27). The intention of having a thicker PSI multilayer in this study was to maximize the transient absorption signal. The negative photocurrent at OCP indicated that cathodic photocurrent was generated (electrons flow from WE via electrolyte to CE), as it was observed in similarly prepared PSI-FTO-based PECs with PSI from different organisms (green alga *Chlamydomonas reinhardtii*, and cyanobacterium *Synechocystis* sp. 6803 [24]) and other constructions based on PSI from *C. merolae* [41,42,43].

**Figure 5:**
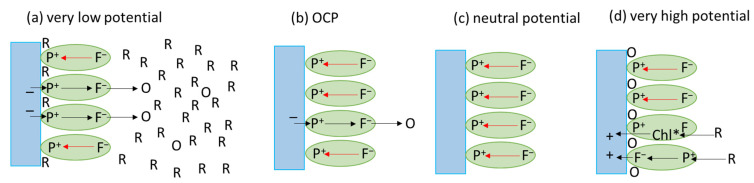
Diagrammatic representation of the ET processes in the PSI-based photoelectrode as a function of the external bias. (**a**) Relatively large cathodic photocurrent due to very low potential applied; (**b**) relatively small cathodic photocurrent at OCP; (**c**) no photocurrent at neutral potential; (**d**) relatively large anodic photocurrent due to a very high potential. Blue rectangles—conducting FTO layer. Green ovals—PSI particles. O and R—oxidized and reduced forms of redox compounds in the electrolyte. Excess of R over O forms in panel (**a**) illustrates the real effect of reduced species domination in bulk electrolyte solution. Black arrows—forward ET, red arrows—back ET. Chl*^—^photoexcited antenna Chls. F—one of the PSI electron acceptors. Note that, for simplicity, only homogeneous orientation of the PSI particles is depicted.

Figure 4b demonstrates that the photocurrent strongly depends on the potential applied to the PSI-FTO photoelectrode. Generally, the lower the potential (lower than OCP), the higher the cathodic (negative) current. On the other hand, at very high positive potentials (points 5 and 6), the anodic current is observed. At moderate potentials (0–220 mV), the photocurrent does not depend on the sequence of preceding potentials (see, e.g., points 2 and 9 in Figure 4b)—identical potentials result in identical photocurrents (similarly points 1 and 4). At a relatively low potential of −180 mV, the photocurrent depends on the sequence of preceding potentials—see points 3 and 7. Qualitatively, similar dependence was reported previously for the PSI-FTO electrodes with PSI isolated from cyanobacterium *Synechocystis* sp. PCC 6803 [27]. A qualitative explanation of this dependence is illustrated in Figure 5. At OCP (red circle in Figure 4b), the photocurrent is low because, in most PSI complexes, charge recombination effectively competes with the transfer of charges outside PSI (Figure 5b). When lowering the potential, the photooxidized primary donors, P^+^, are reduced by electrons delivered from FTO, and, thus, P^+^F_A/B_^−^ → PF_A/B_ recombination becomes less pronounced (Figure 5a). A neutral potential applied to WE, ~(+220 mV) (points 1 and 4 in Figure 4b), completely eliminates photocurrent due to inefficient reduction of P^+^ by FTO and the dominating charge recombination process (Figure 5c). Finally, at the very positive potentials of +420 and +620 mV, close to or higher than the redox midpoint potential of P^+^/P (~450 mV [9,44]), the primary donor P becomes permanently oxidized to P^+^ in a fraction of particles [27], and PSI complexes with such oxidized primary donors become inactivated, i.e., no charge separation can effectively occur and, thus, no photoelectrons can be generated (Figure 5d). In this context, observation of anodic (positive) current may have a double origin (Figure 4b). As proposed previously for different photosynthetic proteins [27,45], one possibility is to assign this current to electrochemical oxidation of photoexcited antenna Chls, which seems likely in the view of very negative redox potential of Chl*/Chl^+^ of −920 mV [45]. An additional source of the anodic photocurrent may be a minor fraction of PSI particles with opposite orientation—with the acceptor side facing the FTO surface and still able to support charge separation (Figure 5d). This fraction may manifest its presence only at high potentials, which inactivate many oppositely oriented PSI particles.

The difference in photocurrents recorded at a relatively low potential (−180 mV, points 3 and 7 in Figure 4b) may be explained by the fact that these two measurements were preceded by very different potentials. Point 3 was recorded after a sequence of moderate potentials (points 1–2), whereas point 7 was recorded just after the very positive potential of +620 mV (point 6) was applied to WE. Most likely, in the latter case, application of the highly oxidizing potential resulted in the oxidization of the redox compounds of the electrolyte (labeled by “O”) present inside the PSI multilayer (Figure 5d). Immediate transition to the very low potential of −180 mV did not provide the full regeneration of the reduced forms (“R”—see Figure 5a). Thus, we conclude that the effect of the low electrode potential on photocurrent in point 7 was decreased by the high potential of the nearby solution (dominated or significantly contributed to by the O form). On the other hand, the effect of the low electrode potential on photocurrent in point 3 was enhanced by the low potential of the nearby solution (dominated by the R form).

We also observed that a further decrease in the applied potential, from −180 mV to −280 mV, results in the decrease rather than the further increase in the cathodic photocurrent (point 8). This effect can be explained by a depletion of the oxidized form of electrolyte, O, in the PSI multilayer. In consequence, electron transfer from F_A/B_^−^ to O was slowed down, the P^+^F_A/B_^−^ → PF_A/B_ recombination was more effective, and cathodic photocurrent decreased.

Finally, it should be emphasized that the photocurrent in the studied system was relatively low: it was ~(−720 nA/cm^2^) at OCP which corresponds to IQE = 0.073%, whereas the maximal cathodic photocurrent of ~(−4600 nA/cm^2^) at −180 mV (point 3 in Figure 4b) translates to IQE = 0.47%. These numbers illustrate that, in both cases, the vast majority of the charge-separated states either recombined or were not formed at all due to the inactivation of the PSI complexes. To further investigate this issue, time-resolved absorption measurements were performed.

### 3.2. Time-Resolved Absorption Measurements

PSI absorption is dominated by antenna Chls, and contribution from ET cofactors is much lower. In the studied PSI particles from *C. merolae*, there are 159 antenna Chls *a* per primary electron donor. From this proportion, using Equation (4), one may estimate that, for the multilayer of PSI particles of A_679_ = 0.27, the maximal expected photobleaching signal resulting from photooxidation of P, ΔA_700_, should be (2–3) mOD:

(4) $$\Delta A_{700}=\frac{A_{679}}{m}\frac{\Delta \varepsilon_{700}\left(P^{+}-P\right)}{\Delta \varepsilon_{679}\left(Chl^{*}-Chl\right)},$$

where *m* = 159 and $\frac{\Delta \varepsilon_{700}\left(P^{+}-P\right)}{\Delta \varepsilon_{679}\left(Chl^{*}-Chl\right)}$ is the ratio of differential molar extinction coefficients at 700 nm related with oxidation of *P* and, at 679 nm, related with excitation of antenna Chls, respectively. This ratio is not exactly known but it may be estimated as ~1–2 given that *P* is the dimer of Chls.

#### 3.2.1. General Comments on the Kinetics of the P^+^ ΔA Signal

To follow the ET reactions in PSI deposited on FTO, we have chosen the probe wavelength of 700 nm. Regarding the millisecond temporal resolution of our measurements, at this wavelength, one may expect observation of immediate appearance of photobleaching signal due to P photooxidation by the excitation laser pulse followed by the decay. In principle, the P^+^ decay signal may be caused by one of the internal recombination reactions or due to ET from an external electron donor to P^+^.

**Figure 6:**
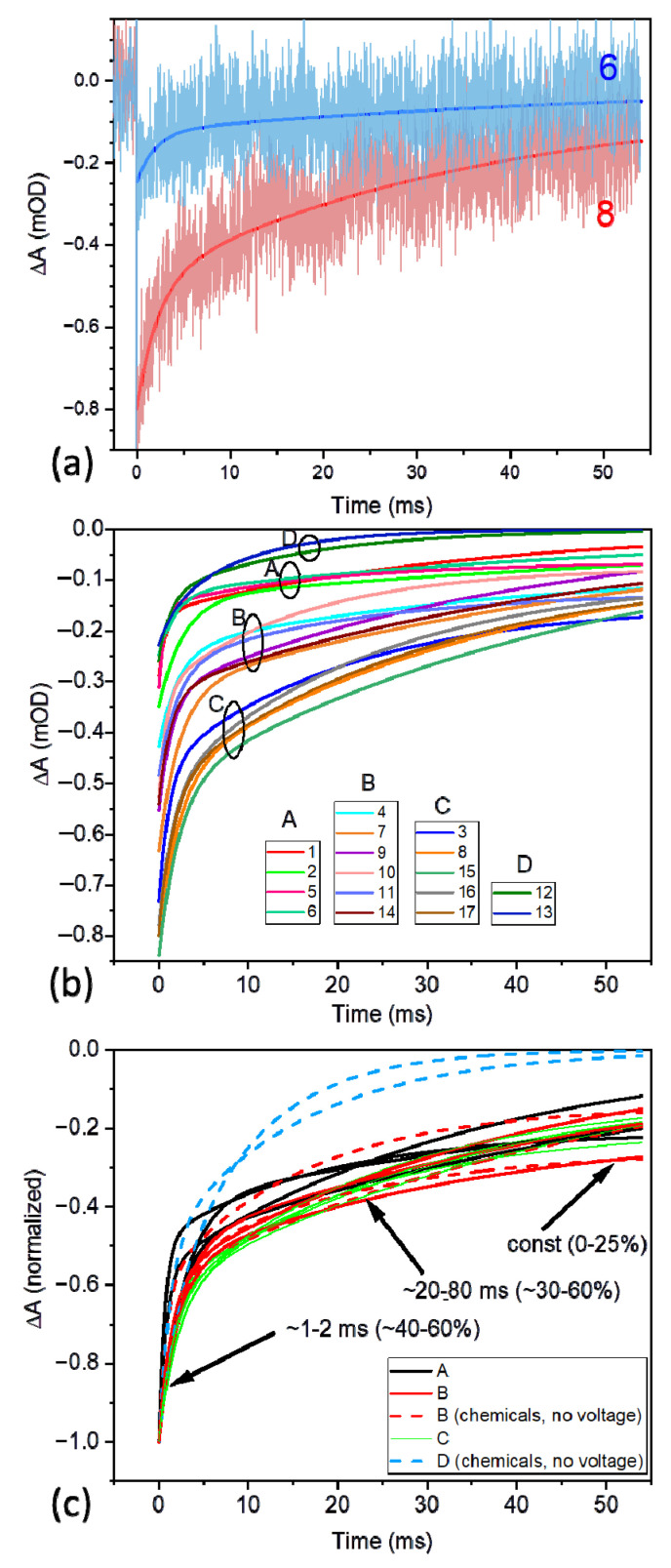
The kinetics of the P700^+^ photobleaching decay at 700 nm in the PSI complexes deposited on FTO glass. (**a**) Examples of experimental kinetics together with fitted bi-exponential curves (fit curves 6 and 8 from Table 1); the region of overlap of the two different kinetic curves is shown in dark pink; (**b**) the fit curves 1–17 from Table 1, lumped into four distinct groups (A–D); (**c**) normalized fit curves 1–17 (groups A-D from panel b are indicated).

Figure 6a presents P^+^ decay kinetics of extreme initial amplitudes and their fits, while Figure 6b presents a collection of fitting curves to the P^+^ decay kinetics recorded at different experimental conditions, including a range of voltages applied to the PSI-FTO electrode, different concentrations of the redox compounds in the electrolyte, and two frequencies of excitation. All these curves are analytically expressed by the bi-exponential function (Equation (3)), and the parameters of this function for each curve are collected in Table 1 (note that section A of Table 1 refers to PSI deposited on FTO, while section B refers to the control/reference experiment performed for PSI dissolved in a buffer solution). The various experimental conditions applied for investigation of FTO-PSI electrode resulted not only in different lifetimes and relative amplitudes of the two exponential phases and the offset (*const*), but also in different initial amplitudes of the photobleaching signal just after the excitation (−ΔA_0_). It is important to notice that, in all cases, the initial (−ΔA_0_) signal was significantly smaller than the expected maximal value of (−ΔA_0,max_) = 2–3 mOD (see above), despite saturating character of the excitation pulses. To estimate the percentage, ΔA_0,rel_, of the PSI particles immobilized on the FTO glass, which supported light-induced charge separation (and, thus, formation of the state P^+^ was observed), we assumed that the same PSI complexes dissolved in an aqueous solution performed charge separation with ~100% efficiency under optimal conditions (saturating excitation and efficient recovery of the photoactive PSI state between the laser flashes by the redox compounds, 10 mM ascorbate and 40 μM DCPIP—see data in section B of Table 1 [24]). Thus, we estimated the values of ΔA_0,rel_ from the following equation:

(5) $$\Delta A_{0,\mathrm{rel}}=\frac{{\Delta A}_{0}}{{\Delta A}_{0}\left(PSI_{aq}\right)}\;f\;100\%,$$

where ΔA_0_ is the initial signal for any measurement (*PSI* in solution or immobilized on FTO slide), ΔA_0_(*PSI_aq_*) is the initial signal for the *PSI* dissolved in the aqueous solution, and *f* is a normalization factor given by Equation (6):

(6) $$f=\frac{A_{679}\left(PSI_{aq}\right)}{A_{679}},$$

where $A_{679}$ is the absorbance of either sample at 679 nm, whereas $A_{679}\left(PSI_{aq}\right)$ is the absorbance of *PSI* particles dissolved in an aqueous solution. The resulting values of ΔA_0,rel_ are collected in Table 1 and were the same as those calculated from Equation (7):

(7) $$\Delta A_{0,\mathrm{rel}}=\frac{{\Delta A}_{0}}{{\Delta A}_{0,\max}}\;100\%$$

for ΔA_0,max_ = −2.4 mOD, which is in a good agreement with the expected range of values of this parameter (2–3 mOD, see above) and corresponds to $\frac{\Delta \varepsilon_{700}\left(P^{+}-P\right)}{\Delta \varepsilon_{679}\left(Chl^{*}-Chl\right)}$ = 1.4.

#### 3.2.2. Initial P^+^ Photobleaching Signal as a Function of Voltage Applied to the PSI-FTO Electrode in the Absence of the Redox Compounds

**Figure 7:**
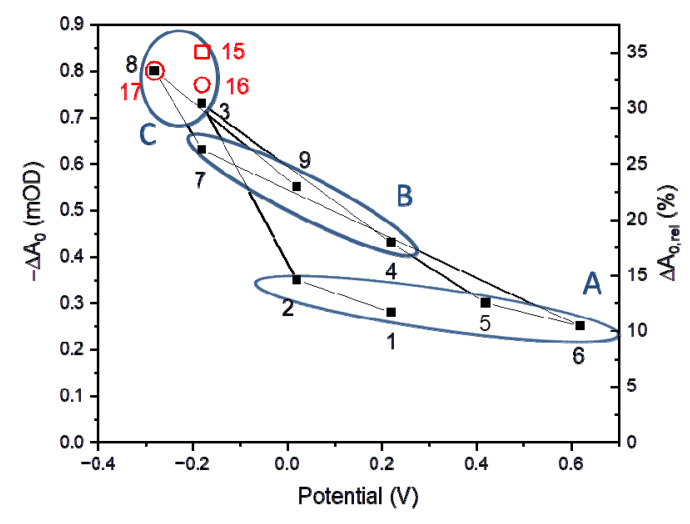
Initial photobleaching amplitudes as a function of voltage applied to PSI-FTO electrode. Data points obtained in the absence of redox compounds in the electrolyte are represented by filled squares (first nine numbered data points from Table 1). Data collected in the presence of 10 mM ascorbate and 200 μM DCPIP are represented by red symbols (data points 15–17 from Table 1). Squares refer to excitation frequency 0.05 Hz, and circles—0.5 Hz. Labels A, B, and C refer to the three distinct groups of kinetics shown in Figure 6b. Left axis—absolute amplitudes, right axis—relative amplitudes.

The first nine measurements of the P^+^ photobleaching kinetics (section A1 of Table 1) were performed at different potentials applied to the PSI-FTO electrode in the absence of any redox compounds in the electrolyte. The sequence of the applied potentials was the same as in the case of photocurrent measurements (Figure 4b). Generally, the lower the potential applied to the WE was, the larger the initial photobleaching signal (−ΔA_0_), similarly as was the cathodic photocurrent. This dependence is presented in Figure 7. The initial signal was expected to be proportional to the size of the fraction of photoactive PSI particles, i.e., particles which were capable of performing charge separation. Apparently, for all potentials, only a fraction of PSI particles was photoactive. This fraction ranged from ~10% at the highest potential (+620 mV, point 6) to ~33% at the lowest potential (−280 mV, point 8). Interestingly, neither of these extreme potentials was fully effective. The extreme positive potential (point 6) should be high enough to fully oxidize P (its midpoint potential is +450 mV) and, thus, inactivate charge separation in all the complexes. However, still, 10% of PSI particles were photoactive, indicating that the electric contact between the FTO substrate and PSI multilayer was imperfect. On the other hand, the extremely low potential of −280 mV increased the fraction of the photoactive complexes from 10% to only ~33%. Thus, two thirds of the PSI complexes remained inactive, either due to inefficient electric contact between the FTO substrate and PSI multilayer or due to structural/functional disturbance of the electrodeposited proteins. One possibility of such disturbance is that the apparently “inactive” PSI complexes performed charge separation, but it was followed by unresolved submillisecond charge recombination events (P^+^A_1_^−^ → PA_1_ and/or P^+^A_0_^−^ → PA_0_) [22,23].

**Figure 8:**
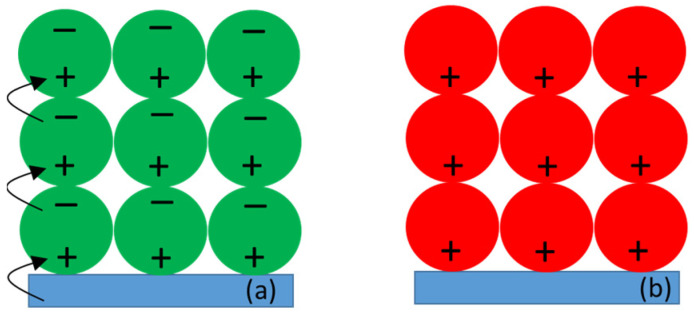
Extreme theoretical voltage effects on the photoactivity of PSI particles in the absence of the redox compounds. (**a**) Low voltage (all PSI complexes contain charge-separated states due to saturating excitation, followed by ET between proteins); (**b**) high voltage (all PSI complexes contain oxidized P). Green circle—photoactive PSI, red circle—inactive PSI, blue rectangle—FTO glass slide. Note that, for simplicity, only homogeneous orientation of the PSI particles is depicted.

Qualitatively, the effect of low and high potential, assuming extreme, unobserved 100% efficiency of each of them, is shown in Figure 8. At low potential, the FTO substrate efficiently donates electrons to P^+^ of the adjacent layer of PSI (Figure 8a). The P^+^ species in the more distant PSI layers can receive the electrons from the final electron acceptor, F_A/B_^−^, of the neighboring protein. This way, the electron may be “transferred” from the FTO surface to the distal PSI layers and recover the ground state of ET cofactors in all the proteins (active state of PSI). Similar electron transfer between neighboring PSI particles forming the multilayer, albeit mediated by highly concentrated redox molecules (5 mM DCPIP and 100 mM ascorbate), was proposed previously [46]. However, since, in the real system, the connection between individual proteins is not so perfect, the electrons from F_A/B_^−^ were not able to reach each PSI complex and this limited the P^+^ re-reduction effect of the low potential. On the other hand, the electrons in F_A/B_^−^ acceptors in such electronically uncoupled PSI particles could either recombine with the hole on P^+^ (recovering the active PSI state) or could be expelled outside PSI—either to oxygen dissolved in the solution or to the buffer molecules [24]. Under such conditions, the PSI particles would adopt the inactive state P^+^F_A/B_, unable to perform charge separation. In the case of high potentials, the delivery of electrons from the FTO glass to PSI was hampered and the described process of PSI inactivation became even more intense (Figure 8b). The observation of the 10% fraction of photoactive PSI complexes under high potential may be explained by the inability of this fraction to transfer electrons outside the protein (see below). Efficient charge recombination could then recover the photoactive ground state PF_A/B_ ready for the next turnover.

Figure 7 shows that the initial P^+^ photobleaching signal (−ΔA_0_) depended not only on the value of the applied potential, but also on the sequence of applied potentials. Points 1, 2, and 7 were obtained for the same potentials as points 4, 9, and 3, respectively, but the former group shows lower photobleaching signals than the respective signals from the latter group. Notably, the latter group of the data points (4, 9, 3) was collected after the application of lower voltages than the respective points in the former group (1, 2, 7). As an example, point 9 was preceded by point 8 measured at −280 mV, whereas point 2 was preceded by point 1 measured at +220 mV. The observed difference in the photobleaching signal at points 9 and 2 (and the two other pairs of points) may be explained by the slow adaptation of the system to the newly applied potentials. This slow adaptation may be rationalized in the following way: after prolonged application of the very negative potential at point 8 (−280 mV), an accumulation of electrons within the multilayer of PSI occurs and partly persists till the measurement of point 9. Thus, the measurement at point 9 is co-determined by the FTO voltage and low potential of the electrons accumulated in the multilayer. The measurement at point 2 was preceded by that at point 1 performed at potential +220 mV, which is much less favorable for the accumulation of electrons in the PSI multilayer. Thus, the measurement at point 2 is co-determined by the FTO voltage and relatively high potential of the solution inside the multilayer. We conclude that the effective potential at point 9 is lower than at point 2. This situation is similar to that described for the photocurrent measurements in chapter 1 (points 7 and 3; Figure 4b), but extended also to higher potentials (points pairs 9/2 and 4/1; compare Figure 4b and Figure 7), likely due to the absence of the redox compounds in the time-resolved absorption experiments (Figure 7).

#### 3.2.3. Kinetics of the P^+^ Photobleaching Signal at Different Voltages Applied to the PSI-FTO Electrode in the Absence of the Redox Compounds

**Figure 9:**
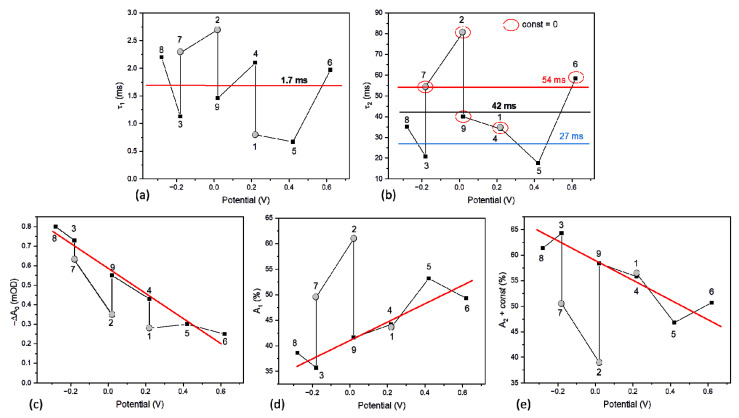
Dependence of bi-exponential fit parameters on voltage applied to the PSI-FTO electrode (with no chemicals added; experiments no. 1–9 in Table 1). (**a**) Lifetime τ_1_ with the average value indicated; (**b**) lifetime τ_2_ with the average values indicated separately for the kinetics fitted with *const* = 0 (54 ms; points in the red ellipses), *const* ≠ 0 (27 ms; remaining points), and for all kinetics (42 ms); (**c**) initial amplitude, −ΔA_0_; (**d**) amplitude A_1_ of the fast phase (~1.7 ms); (**e**) the sum of the amplitude A_2_ of the slow phase (~42-ms) and *const* parameter. The points shown as gray circles (1, 2, 7) are those for which the initial amplitude (−ΔA_0_; panel (**c**)) deviates from the general trend due to specific sequence of the applied potentials. The red lines in panels c-e indicate trends in the values of the amplitudes with neglected points 1, 2, and 7.

The overall shapes of the kinetic curves from the measurements 1–9 were observed to be not very different from one another (Figure 6c). A common feature of all the kinetic traces is their bi-exponential decay character. Additionally, in some kinetic measurements, a nondecaying component (*const*) was resolved, and, in some, not. The fit parameters are presented in section A1 of Table 1 and, in a graphic way, in Figure 9. The faster decay phase lifetime, τ_1_, of the value ranging from ~0.5 ms to ~2.5 ms did not show any systematic dependence on the voltage applied to the PSI-FTO electrode (Figure 9a), and its mean value was 1.7 ms. The slower decay component (τ_2_) ranged from ~20 to ~80 ms, again without systematic dependence on the voltage (Figure 9b). The kinetics fitted with the parameter *const* = 0, yielded, as expected, a somewhat larger lifetime τ_2_ of the mean value 54 ms. When the *const* parameter was non-zero, the mean value of τ_2_ was 27 ms, and the overall mean lifetime τ_2_ was 42 ms. The broad distribution of the two lifetimes is assigned to a limited signal-to-noise ratio. The mean value of 1.7 ms fits very well the lifetime of the P^+^F_X_^−^ → PF_X_ recombination [22,23], whereas the mean values 27–42–54 ms fit the lifetime value of the P^+^F_A/B_^−^ → PF_A/B_ recombination [22,23]. The *const* parameter (of the relatively small value of up to 0.2, see Table 1) may reflect a fraction of PSI particles with particularly long-lived P^+^ state. Such long-lived state may be caused by (1) particularly slow P^+^F_A/B_^−^ → PF_A/B_ recombination (reported to be 86 ms in [22]) that was not properly resolved in the 54-ms time window of the experiment, and (2) escape of the electron from F_A/B_^−^ outside PSI, leaving the complex in the state P^+^F_A/B_ [24]. The observation that the fits with non-zero *const* parameter resulted in significant acceleration of the slower decay component (on average from 54 to 27 ms) indicates that a large fraction of the *const* value was due to the former reason.

**Figure 10:**
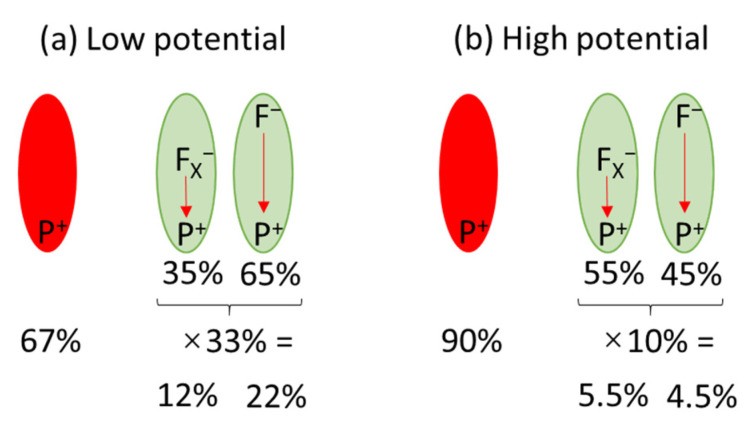
Fractions of active (green) and inactive (red) PSI complexes under (**a**) low (−280 mV) and (**b**) high (+620 mV) potential. Within active PSI complexes, fractions recombining from the states P^+^F_X_^−^ and P^+^F_A/B_^−^ are shown.

Despite overall similar shape and lifetimes of all the kinetic curves (1–9) and their limited signal-to-noise ratio, detailed analysis of the amplitudes of the fast and slow decay phases revealed their systematic dependence on the applied voltage and their correlation with the voltage dependence of the initial photobleaching signal (Figure 9c–e). As noted above, initial photobleaching amplitude increases with decreasing potential (Figure 9c), which is correlated with the decrease in the amplitude of the fast decay component, from ~55 to ~35% (Figure 9d), and an increase in the amplitude of the slow decay from ~45 to ~65% (Figure 9e). Since, with the given signal-to-noise ratio and time window of the experiment, it was impossible to properly separate the amplitudes of the slow exponential and *const* components, these two were lumped together (Figure 9e). The observed increased contribution of the slow decay component (Figure 9e) with lowering the voltage, paralleled by an increase in the total signal (Figure 9c), was as expected. A similar effect was reported for PSI suspended in an aqueous solution upon increasing its reducing strength controlled by the concentration of redox chemicals (instead of the application of low voltage to the electrode; see [24]). Low potential reactivates preferentially PSI complexes that are able to perform full charge separation (PF_A/B_ → P^+^F_A/B_^−^), since only such complexes are expected to be in the inactive state P^+^F_A/B_. On the other hand, PSI complexes unable to perform charge separation beyond P^+^F_X_^−^ are not expected to form the inactive state P^+^F_A/B_, since the P^+^F_X_^−^ → PF_X_ recombination seems to be prevalent over the escape of the electron from F_X_^−^ outside the protein. In that context, the fraction of the PSI complexes with charge separation limited to the state P^+^F_X_^−^ should not increase at all upon lowering the potential. However, as shown in Figure 10 (collecting the data from Table 1 (section A1) and Figure 9), this fraction also increases (from ~5.5% to ~12%), albeit to a lesser extent than the fraction showing full charge separation (an increase from ~4.5% to ~22%). A possible explanation of this phenomenon may be that the fast, ~1.7 ms, component is contributed to by fast electron donation to P^+^ from the FTO electrode.

#### 3.2.4. Kinetics of the P^+^ Photobleaching Signal in the Presence of the Redox Compounds

Section A2 of Table 1, which includes the results obtained without any potential applied to the photoelectrode but with the addition of different concentrations of ascorbate and DCPIP, shows that the re-reduction of P^+^ by the used redox compounds is less efficient than the re-reduction of P^+^ by the low voltage applied to the WE. The initial photobleaching signal, whose amplitude (ΔA_0,rel_) is a measure of this efficiency, is limited to ~20–23% in the former case compared to ~33% in the case of the lowest potential applied. Unexpectedly, the addition of 4–200 μM DCPIP, which in solution accelerates P^+^ re-reduction by ascorbate [3,22,24,39], exerts no effect on ΔA_0,rel_ compared to 10 mM ascorbate alone (datasets 10, 11, 14 in Table 1; group B in Figure 6b,c). On the other hand, decreasing the excitation frequency from 0.05 Hz to 0.5 Hz decreases the initial photobleaching signal twice to 10–11% (datasets 12–13 in Table 1; group D in Figure 6b,c). This demonstrates that ascorbate needs significantly more than 2 s to re-reduce P^+^ in the PSI multilayer, which agrees with the previous reports [24,39]. The shapes of the kinetic curves measured with 0.05 Hz excitation resembled those measured without redox compounds and were fitted by similar fast (~1–2 ms, ~40%) and slow (~20–50 ms, ~60%) decay components, assigned again to the P^+^F_X_^−^ → PF_X_ and P^+^F_A/B_^−^ → PF_A/B_ recombination events, respectively. Significantly faster decay was observed upon 0.5 Hz excitation frequency (datasets 12–13 in Table 1; group D in Figure 6b,c), assigned to ~10% fraction of PSI particles permanently photoactive due to efficient, mostly P^+^F_X_^−^ → PF_X_, charge recombination.

**Figure 11:**
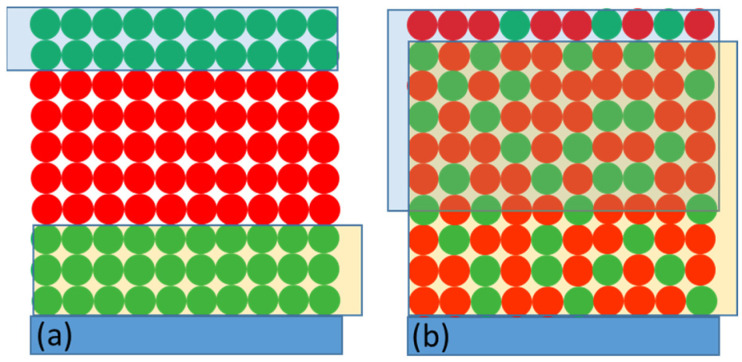
(**a**,**b**) Two models of the active (green) vs. inactive (red) PSI particles distribution across their multilayer on the FTO substrate. Semi-transparent yellow and blue rectangles are the areas of the effective P^+^ re-reduction by the low voltage (−180 mV) and by redox compounds (10 mM ascorbate and 200 μM DCPIP), respectively.

Section A3 of Table 1 shows the results of experiments performed both with negative potentials applied to WE and redox compounds in the electrolyte. Combination of the low potential (−180 mV) and presence of 10 mM ascorbate and 200 μM DCPIP in the electrolyte increased the initial photobleaching signal, ΔA_0,rel_, merely by 5%, from 30 to 35%, compared to the effect of the low potential alone (compare datasets 3 and 15 in Table 1 and in Figure 7). Apparently, the effects of the P^+^ re-reduction by low potential alone (ΔA_0,rel_ ≈ 30%; dataset 3) and redox compounds alone (ΔA_0,rel_ ≈ 20%; datasets 10, 11, 14) are not additive, as could have been expected considering limited penetration depths of the electric field from one side and the redox compounds from the opposite side of the PSI layer. Such a hypothetical situation is presented in Figure 11a, whereby it is assumed that all PSI particles in the multilayer are potentially photoactive, but their middle region becomes inactive (the state P^+^F_A/B_ is formed) due to the limited access of the two reducing agents. To reconstruct the experimentally observed fractions of photoactive PSI complexes (~30% for low potential alone (−180 mV), ~20% for redox compounds alone, and ~35% for combinations of both), an alternative model was developed (Figure 11b; see SI for details). In this model, the small enhancement effect of the combination of the low potential and redox compounds was obtained by assuming limited but large penetration depth of both these agents, and homogenous distribution of photoactive and inactive PSI complexes across the multilayer. The resulting fraction of the PSI complexes inactive even in the presence of re-reducing agents was 65%, and the penetration depths of the low voltage and the redox compounds were 90% and 65% of the multilayer thickness, respectively. The penetration depth of the redox compounds limited to 65% is in contrast with PSI multilayer being fully permeable for the redox molecules (5 mM DCPIP and 100 mM ascorbate), concluded in [46], despite the thickness in their study, 1–2 μm, being larger than in ours (40 × ~(15 nm) ≈ 0.6 μm). This difference can be related with different methods of PSI deposition and/or different concentrations of DCPIP and mM ascorbate.

Increasing the excitation frequency from 0.05 to 0.5 Hz, in the presence of both low voltage and redox compounds (points 15 and 16, respectively, in Figure 7), decreased ΔA_0,rel_ signal only slightly. This small change indicates that the P^+^ re-reduction effect of the low voltage is significantly faster than that of the used redox compounds (see above).

It is interesting to note that similar studies of the P^+^ decay performed for the same preparation of *C. merolae* PSI but suspended in a buffer solution containing 10 mM ascorbate and up 40 μM DCPIP revealed photoactivity of all PSI particles, even at 0.5 Hz excitation frequency (ΔA_0,rel_ = 100%; see part B of Table 1 and [24]), whereas only ~20–35% of immobilized PSI are active in the presence of the same redox compounds. The unsolved question arises: what is the nature of the remaining major fraction of inactive immobilized PSI complexes? Do they constitute a “rogue” fraction of PSI particles in the state P^+^F_A/B_ without efficient electric connection with the re-reducing agents, do they perform fast unresolved charge recombination, or are they disturbed in a more profound way, e.g., by the electrodeposition procedure? The latter possibility seems to be least likely, since the femtosecond studies on cyanobacterial PSI electrodeposited on the FTO glass revealed that the excitation energy transfer and primary charge separation were similar to those for PSI in solution [27].

### 3.3. Comparison of the Time-Resolved Absorption and Photocurrent Results

Table 2 presents the main results obtained from the time-resolved absorption and photocurrent experiments for the FTO-PSI system. Moreover, the results obtained for this system are compared with those reported previously for *C. merolae* PSI complexes suspended in a buffer solution containing ascorbate and DCPIP [24]. Time-resolved absorption data show fractions of PSI able to perform charge separation and, in principle, able to transfer the electron outside PSI. On the other hand, photocurrent data show the electrons successfully expelled from PSI to the external electric circuit of the PEC. The first observed effect of PSI immobilization on FTO is a decrease in the fraction of proteins able to perform charge separation from 100% in solution to 20–35% after deposition on FTO, depending on the potential applied (OCP or −180 mV) and redox compounds added. The second effect of the PSI immobilization on FTO is a decrease in the fraction of proteins able to perform full charge separation (formation of the P^+^F_A/B_^−^ state) from ~90% in solution to 10–20% after deposition on FTO, depending on the potential applied. The third effect, an ability to expel the electron outside PSI, was not directly determined for the FTO-PSI system. If one assumes that this ability is the same as in the solution (~2% [24]), the resulting fraction of PSI performing ET outside PSI would be 0.2–0.4%, which is a range of values similar to IQE (0.073–0.47%). In such a scenario, one may conclude that the internal charge recombination (occurring between F_A/B_^−^ and P^+^ in an individual PSI complex) was the major factor responsible for the very limited IQE measured in the studied system. Alternatively, the fraction of PSI showing ET outside the protein may be higher in the film than in the solution. This may happen since the ET from the FTO electrode to P^+^ may be faster than from the redox compounds in solution, as suggested by the results discussed above. In such a case, the external charge recombination, i.e., ET from F_A/B_^−^ in one PSI complex to P^+^ in another one, would be the third important factor – in addition to limited photoactivity of PSI and internal charge recombination – responsible for the low IQE value.

## 4. Summary and Conclusions

Our studies demonstrate that electrodeposition of PSI complexes on FTO significantly affects their ability to support full natural forward electron transfer. Up to 90% of immobilized PSI particles remain inactive. This number may be reduced to 65% by application of reducing agents, such as low electric potential applied to the FTO-PSI photoelectrode, or addition of redox mediators to the electrolyte solution being in contact with the protein film. This work indicates that (quasi)permanent photo-oxidation of PSI complexes (PSI in the state P^+^F_A/B_) may be the reason for their decreased photoactivity, and that it may originate from limited access of the reducing agents to a fraction of crowded protein particles. Additional causes of PSI inactivation may be fast, unresolved charge recombination (P^+^A_1_^−^ → PA_1_ and/or P^+^A_0_^−^ → PA_0_) or other types of ET disturbance. On the other hand, the fraction of photoactive complexes is inhomogeneous—roughly half of the active PSI do not perform full charge separation, but they undergo P^+^F_x_^−^ → PF_x_ charge recombination. Despite significant perturbation of forward ET inside immobilized PSI particles, it is not the major reason for the low, <0.5%, photon-to-photocurrent efficiency in the studied system. Apparently, the P^+^F_x_^−^ → PF_x_ and P^+^F_A/B_^−^ → PF_A/B_ charge recombination reactions are generally much more effective than the electron transfer from FTO to PSI and from PSI to the counter electrode in the studied system. We conclude that, in designing new efficient PSI-based photoelectrodes, it is essential to provide both intact operation of PSI and efficient electron and hole uptake from PSI by artificial elements of the solar conversion system.

## Figures and Tables

**Table 1:** Experimental conditions and parameters of transient absorption kinetics of *C. merolae* PSI complexes recorded at 700 nm. (A) PSI complexes deposited on FTO; (B) PSI suspended in aqueous solution ^(1)^.

Expe-riment Number	Experimental Conditions				Parameters of Kinetic Traces					
	Potential (V)	[Asc] (mM)	[DCPIP] (µM)	f (Hz)	−ΔA_0_ (mOD)	ΔA_0,rel_ (%)	τ_1_ (ms) A_1_	τ_2_ (ms) A_2_	*const*	τ_ave_ (ms)
(A) PSI-FTO, A_679_ = 0.27										
(A1) Experiments with variable potential of WE and without redox compounds in electrolyte										
1	0.22 ^(2)^	-	-	0.05	0.28	12	0.8 0.44	34 0.56	0.00	19.4
2	0.02	-	-	0.05	0.35	15	2.7 0.61	81 0.39	0.00	33.2
3	−0.18	-	-	0.05	0.73	31	1.1 0.36	21 0.44	0.20	-
4	0.22	-	-	0.05	0.43	18	2.1 0.44	34 0.36	0.20	-
5	0.42	-	-	0.05	0.30	13	0.7 0.53	18 0.29	0.18	-
6	0.62	-	-	0.05	0.25	10	2.0 0.49	59 0.50	0.01	30.5
7	−0.18	-	-	0.05	0.63	26	2.3 0.50	55 0.50	0.00	28.7
8	−0.28	-	-	0.05	0.80	33	2.2 0.39	35 0.56	0.05	-
9	0.02	-	-	0.05	0.55	23	1.5 0.42	40 0.58	0.00	23.8
(A2) Experiments with no potential applied to WE and with redox compounds in electrolyte										
10	-	10	-	0.05	0.52	22	0.8 0.41	16 0.46	0.13	-
11	-	10	4	0.05	0.48	20	2.1 0.44	21 0.31	0.25	-
12	-	10	40	0.5	0.26	11	1.3 0.50	15 0.50	0.00	8.2
13	-	10	200	0.5	0.23	10	2.0 0.29	9.4 0.71	0.00	7.3
14	-	10	200	0.05	0.54	23	1.4 0.41	49 0.59	0.00	29.5
(A3) Experiments with negative potentials applied to WE and with redox compounds in electrolyte										
15	−0.4	10	200	0.05	0.84	35	2.2 0.39	47 0.61	0.00	29.5
16	−0.4	10	200	0.5	0.77	32	1.6 0.34	24 0.55	0.11	-
17	−0.5	10	200	0.5	0.80	33	1.6 0.36	29 0.54	0.10	-
**(B) PSI in solution, A_679_ = 0.6 ^(3)^**										
	-	10	40	0.5	5.3	100	6.3 0.12	41 0.86	0.02	37

Amplitudes A_1_, A_2_ and *const* were normalized: A_1_ + A_2_ + *const* = 1. ΔA_0,rel_ is the relative initial transient absorption signal just after the excitation. The average decay time was estimated from the equation τ_ave_ = τ_1_A_1_ + τ_2_ A_2_, only for kinetics with *const* ≈ 0. ^(1)^ Results taken from [24]. ^(2)^ No potential was applied, but the measured potential of the solution was 0.22 V. ^(3)^ A_679,0.6 cm_ = 0.6 is the absorbance of the PSI solution of 6 mm thickness (defined by the diameter of the excitation beam) of A_679,1cm_ = 1.

**Table 2:** Comparison of parameters related to charge separation in *C. merolae* PSI, ET outside PSI, and IQE estimated from the time-resolved absorption or photocurrent experiments.

Type of Experiment	Parameter	In Solution ^(3)^	Immobilized on FTO			
			No Redox Compounds Added		+10 mM Ascorbate +200 μM DCPIP	
			@OCP (+115 mV)	@low Potential (−180 mV)	@OCP (+115 mV)	@low Potential (−180 mV)
Time-resolved absorption	Fraction of photoactive PSI ^(1)^	100%	20%	31%	-	35%
	Fraction of fully photoactive PSI ^(2)^	~90%	~10%	~20%	-	~20%
	Fraction of PSI showing ET outside PSI	2%	(0.2%)^(4)^	(0.4%) ^(4)^	-	(0.4%) ^(4)^
Photocurrent	IQE	-	-	-	0.073%	0.47%

^(1)^ PSI complexes undergoing any of the charge separation steps; ^(2)^ PSI complexes undergoing full charge separation yielding the final state P^+^F_A/B_^−^; ^(3)^ data from [24]; solution contained 10 mM ascorbate and 40 μM DCPIP; ^(4)^ the values in brackets were estimated assuming 2% efficiency of ET outside immobilized PSI, i.e., the efficiency measured for PSI complexes suspended in solution.

## Data Availability

The data that support the findings of this study are available from the corresponding authors upon a reasonable request.
